# Aquaporins in Brain Tumor-Related Edema: A Narrative Review

**DOI:** 10.1159/bmh/aboag004

**Published:** 2026-09-05

**Authors:** Yusor Al-Nuaimy, Denise Baloi, Clayton Rawson, Jerick Kim, Michael Karsy, Mehrdad Pahlevani

**Affiliations:** a College of Medicine, Ajman University, Ajman, United Arab Emirates; b College of Human Medicine, Michigan State University, Lansing, MI, USA; c College of Medicine, Noorda College of Osteopathic Medicine, Provo, UT, USA; d School of Medicine, Wayne State University, Detroit, MI, USA; e Department of Neurosurgery, University of Michigan, Ann Arbor, MI, USA; f Department of Neurosurgery, Jamaica Hospital Medical Center, New York, NY, USA

**Keywords:** Aquaporin-4, Brain tumor, Peritumoral edema, Blood-brain barrier, Glymphatic system

## Abstract

**Background:**

Brain tumor-related edema is a major contributor to morbidity in patients with intracranial neoplasms, arising from interactions between blood-brain barrier (BBB) dysfunction, tumor biology, and dysregulated water transport. Aquaporins (AQPs), and particularly AQP4, are central to cerebral water homeostasis, and alterations in their expression and spatial polarization within and around brain tumors influence both cytotoxic and vasogenic edema. This narrative review synthesizes contemporary evidence on AQP biology in the tumor-bearing brain and critically appraises the translational maturity of AQP-directed therapy.

**Summary:**

AQP4 is the predominant central nervous system AQP, concentrated at astrocytic end-feet and essential for water flux across the BBB and along glymphatic pathways. In high-grade gliomas, AQP4 is upregulated and loses perivascular polarity, a pattern that accompanies extensive vasogenic edema, impaired perivascular clearance, and enhanced tumor cell invasion. Low-grade gliomas and meningiomas show more restricted or vascular-predominant AQP expression, including AQP1 in meningioma vasculature, and comparatively less peritumoral edema. Experimental work demonstrates a bidirectional, context-dependent role for AQP4: it promotes astrocytic swelling when the BBB is intact, yet facilitates reabsorption of extracellular fluid once the barrier is disrupted. This duality has not been resolved in human tumor tissue and constrains any single-direction therapeutic strategy. Candidate interventions, including small-molecule inhibitors (AER-270/AER-271), monoclonal antibodies, SUR1–TRPM4 modulation, RNA interference, and CRISPR/Cas9-based editing, have shown activity in rodent injury models but have not been evaluated in brain tumor populations.

**Key Messages:**

(1) AQP4 is not uniformly pro-edematous; its net effect depends on BBB integrity, edema phase, and preservation of perivascular polarity, so undifferentiated inhibition risks impairing fluid clearance in precisely the tumors that most need it. (2) The evidence base remains dominated by rodent injury models and small immunohistochemical series rather than human tumor tissue, and no AQP-targeted agent has entered a brain tumor trial. (3) Meaningful progress will require human-tissue studies that quantify AQP4 polarity rather than total expression, prospective imaging biomarkers such as diffusion tensor image analysis along the perivascular space capable of stratifying patients before treatment, and astrocyte-targeted, temporally controlled gene-editing approaches rather than constitutive gene deletion.

## Introduction

Brain tumor-related edema is a major contributor to morbidity and mortality in patients with intracranial neoplasms, often exerting greater clinical impact than the tumor mass itself. Peritumoral edema is predominantly vasogenic in nature [1, 2], arising from blood-brain barrier (BBB) breakdown due to tumor-induced inflammation, hypoxia, and angiogenic signaling. This process permits plasma ultrafiltrate to accumulate within the extracellular space, particularly in the white matter [3, 4]. BBB disruption is especially pronounced in high-grade gliomas and many metastatic lesions, where leaky neovasculature and extracellular matrix degradation amplify extracellular swelling [5]. Distinguishing cytotoxic from vasogenic edema remains clinically important as these entities differ in reversibility and therapeutic responsiveness; however, mixed phenotypes frequently coexist within the tumor microenvironment [6, 7].

Aquaporin-4 (AQP4), the most abundant AQP in the brain, is enriched at astrocytic end-feet adjacent to microvessels and the pia mater, where it facilitates rapid, bidirectional water transport essential for neurovascular coupling, extracellular ion regulation, and glymphatic clearance [8]. Its polarized distribution is mediated by the M1 and M23 isoforms, which assemble into orthogonal arrays of particles (OAPs) that influence both membrane permeability and cellular localization [9, 10]. Additional AQPs, including AQP1, AQP3, AQP5, and aquaglyceroporins such as AQP9, contribute to cerebrospinal fluid (CSF) dynamics, metabolic water flux, and astrocyte-neuron communication [11].

In the tumor setting, AQP expression and localization become substantially dysregulated. Tumor-associated hypoxia, cytokine signaling, extracellular matrix remodeling, and glutamate-mediated excitotoxicity collectively modulate AQP expression, often producing excessive or mislocalized AQP4 that enhances water permeability and exacerbates edema formation [12, 13]. In high-grade gliomas, AQP4 is frequently upregulated and redistributed away from perivascular end-feet toward the tumor core, disrupting normal perivascular drainage and amplifying both cytotoxic and vasogenic components of edema [14]. By contrast, meningiomas and low-grade gliomas exhibit different AQP profiles, with some tumors showing elevated AQP1 correlated with vasogenic edema volume, while others show minimal AQP upregulation despite substantial mass effect [15]. Brain metastases, particularly those arising from lung, breast, and melanoma primaries, also display altered AQP4 expression associated with peritumoral fluid accumulation and BBB instability [16, 17].

Current therapies, including corticosteroids, osmotic agents, and anti-angiogenic drugs, remain only partially effective because they do not directly target the molecular machinery governing water flux [3, 18, 19]. This gap has prompted increasing interest in AQPs as therapeutic targets, with experimental strategies including small-molecule inhibitors, monoclonal antibodies, gene-editing approaches, and modulation of AQP4–OAP assembly [20–23]. Enthusiasm for these strategies, however, has outpaced the evidence supporting them, and much of the literature restates the same small set of rodent experiments without reconciling their contradictions.

### Scope and Approach

This article is a narrative review. Relevant literature was identified through searches of PubMed, Google Scholar, and Scopus covering 2000–2025, using combinations of the terms AQP, AQP4, AQP1, brain tumor edema, cytotoxic edema, vasogenic edema, glioma, meningioma, metastases, glymphatic system, and AQP4 inhibitors, supplemented by hand-searching of reference lists. We deliberately adopted a qualitative rather than a quantitative synthesis. The available evidence is dominated by heterogeneous small-animal experiments and immunohistochemical case series that differ in species, injury paradigm, tumor histology, antibody and scoring method, and in whether AQP4 is reported as total staining intensity or as degree of perivascular polarization. These outcomes are not expressed on a common scale, and no two studies define the peritumoral region identically; pooling them would generate a spuriously precise summary estimate rather than a more reliable one, and would obscure the very finding that matters most, namely, that AQP4 manipulation produces directionally opposite effects in cytotoxic and vasogenic models. A qualitative synthesis instead allows these opposing results to be placed side by side and interrogated. The review is organized around AQP structure and physiology, barrier disruption and edema classification, tumor-specific dysregulation, imaging correlates with potential clinical utility, current and emerging therapy, and a critical appraisal of what remains unresolved.

## AQP Structure, Isoforms, and Central Nervous System Physiology

AQPs are small (∼30 kDa) integral membrane proteins composed of six transmembrane helices and two short half-helices that converge to form a narrow water-conducting pore. These proteins assemble as tetramers within the membrane, with each monomer functioning as an independent water channel [24]. Each monomer contains conserved NPA motifs (asparagine-proline-alanine) and an aromatic/arginine (ar/R) selectivity filter, which together confer high specificity for water while excluding ions and larger solutes [10].

AQP4 is the predominant AQP of the central nervous system, with high expression in astrocytes lining the BBB, glia limitans, and ependymal and ventricular surfaces, where it mediates water exchange between blood, CSF, and parenchyma [8, 24]. Deletion of AQP4 reduces astrocyte plasma membrane water permeability approximately sevenfold, underscoring its central role in cerebral water transport [8, 24]. AQP1 is expressed in the choroid plexus epithelium, where it contributes to CSF secretion, whereas AQP9 is found in subsets of astrocytes and may facilitate lactate or glycerol transport under metabolic stress [8, 11]. AQP4 expression is dynamically regulated across multiple pathologies, including contusion, meningitis, tumor infiltration, and subarachnoid hemorrhage, and its functional consequence, whether promotion of edema formation or facilitation of fluid clearance, is highly disease dependent [8].

AQP4 exists as two principal splice isoforms, M1 and M23, which differ in their N-terminal sequences and assemble into distinct supramolecular configurations known as OAPs [10, 25]. M23 homotetramers form large, relatively immobile OAPs that stabilize AQP4 polarization at perivascular end-feet, whereas M1 homotetramers form smaller or fewer arrays and exhibit greater membrane mobility [10, 25]. This increased mobility has been proposed to facilitate glioma cell migration, and the M1:M23 ratio, therefore, represents a key determinant of AQP4 organization and localization within astrocytes. Mutational studies of loop D demonstrate that tetramerization is not required for baseline water permeability but is essential for tonicity-dependent relocalization of the channel [10, 25].

Polarized localization of AQP4 to the astrocytic end-foot is maintained by an anchoring complex that includes the dystrophin-associated protein assembly and α-syntrophin; disruption of these anchoring partners produces mislocalization of AQP4 away from the perivascular membrane without necessarily reducing total AQP4 protein. Under conditions of central nervous system injury, AQP4 may also shift its interaction profile from transient receptor potential vanilloid 4 (TRPV4) under physiologic conditions toward the sulfonylurea receptor 1–transient receptor potential melastatin 4 (SUR1–TRPM4) channel complex, contributing to coordinated ion and water influx and to the development of cytotoxic edema [10, 22]. This distinction between total expression and spatial polarization is not a technical detail: it is the single most important source of apparent contradiction in the literature because studies that report only staining intensity cannot distinguish an astrocyte with abundant, correctly polarized AQP4 from one with abundant but depolarized channel.

## BBB Disruption in Brain Tumors

Brain tumor-related edema arises primarily from disruption of the blood-tumor barrier, the pathological analog of the BBB. The BBB is formed by non-fenestrated cerebral endothelial cells connected by tight junctions [26, 27]. These endothelial cells differ from peripheral capillaries in that they contain relatively few endocytic vesicles, thereby limiting transcellular transport. The barrier is further supported by astrocytic end-feet processes that envelop cerebral microvessels and contribute to its structural and functional integrity [27]. The presence of brain tumors increases BBB permeability primarily through disruption of inter-endothelial tight junctions and diminished astrocytic support. In high-grade gliomas and metastatic lesions, tumor-associated vasculature often lacks tight junctions and tends to exhibit characteristics of the parent tissue, including expression of peripheral endothelial markers such as AQP1 [11, 26].

Tumor-derived angiogenic factors, including vascular endothelial growth factor (VEGF) and scatter factor/hepatocyte growth factor, promote phosphorylation and downregulation of key tight junction proteins such as occludin, claudin-1, claudin-5, and ZO-1 [28]. These molecular alterations contribute to the increased vascular permeability seen in brain tumor-related edema. In contrast, corticosteroids exert therapeutic effects in part by promoting dephosphorylation of occludin and ZO-1, thereby decreasing endothelial permeability. Additionally, rapidly proliferating tumors frequently outgrow their vascular supply, resulting in hypoxia that further amplifies VEGF production and perpetuates barrier disruption (Fig. 1).

**Fig. 1.**
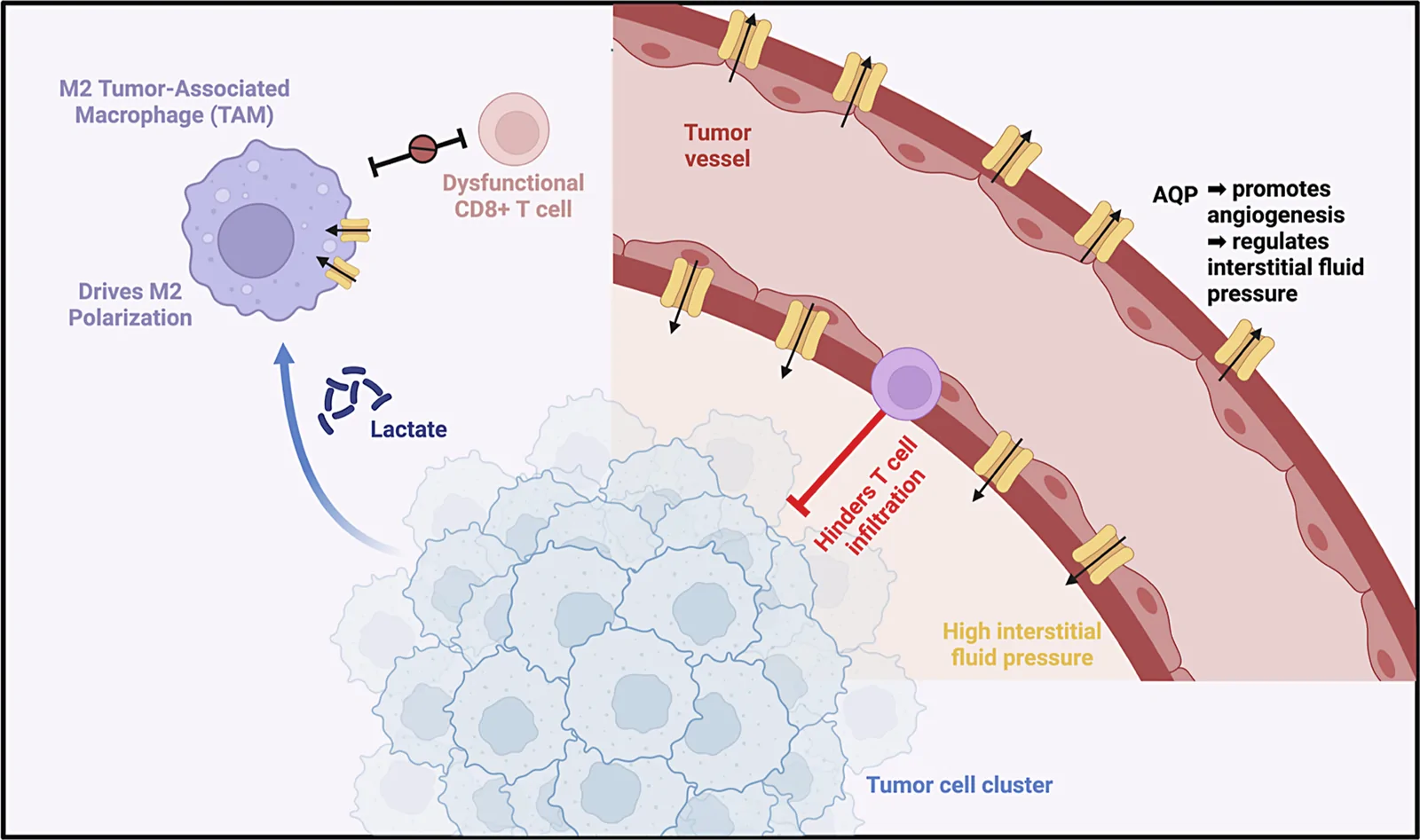
Lactate released by the tumor cell cluster promotes polarization of macrophages toward an M2 tumor-associated macrophage phenotype, which contributes to CD8^+^ T-cell dysfunction. Aquaporin (AQP) channels expressed in the tumor vasculature support angiogenesis and regulate fluid movement, contributing to elevated interstitial fluid pressure. Increased interstitial pressure creates a physical barrier that limits infiltration of T cells from the circulation into the tumor. Arrows indicate the direction of the depicted effects, whereas blunt-ended lines indicate inhibition or impairment.

## Classification and Pathophysiology of Tumor-Related Edema

Klatzo first classified cerebral edema as cytotoxic or vasogenic in 1967 [29], a framework later expanded by Fishman in 1975 to include interstitial edema [30]. Cytotoxic edema refers to intracellular swelling resulting from ionic imbalance, typically due to failure of ATP-dependent ion pumps, leading to sodium and water influx into glial, neuronal, and endothelial cells [1]. This form is most often observed in the early stages of cerebral ischemia and hypoxia, during which the BBB remains intact. In contrast, vasogenic edema arises from disruption of the BBB, resulting in increased permeability of cerebral endothelial cells to albumin and other plasma proteins. Although these categories are conceptually distinct, they are not mutually exclusive; cytotoxic and vasogenic edema frequently coexist, and one form may evolve into the other over time [19]. Accordingly, while brain tumors most commonly produce vasogenic edema, this pattern is not exclusive, and the dichotomy is better understood as a spectrum whose composition shifts over the natural history of the lesion and with treatment.

## AQP4 in Cytotoxic versus Vasogenic Edema: A Context-Dependent Role

Because astrocytes are the principal cells undergoing swelling in cytotoxic edema and the main site of AQP4 expression, AQP4 has long been assumed to play an integral part in cytotoxic brain edema [31, 32]. Consistent with this, AQP4 deletion reduces brain swelling and improves neurological outcome in models of water intoxication and focal cerebral ischemia [33], reduces encephalopathy and brain edema in experimental acute liver failure [31], and, when combined with deletion of TRPV4, largely prevents acute cytotoxic edema after ischemic injury [34]. Conversely, transgenic glial overexpression of AQP4 accelerates cytotoxic brain swelling [35]. Taken together, these experiments make a reasonably coherent case that AQP4 provides the conduit for astrocytic water entry when the barrier is intact.

The picture reverses in vasogenic edema. Papadopoulos and colleagues demonstrated that AQP4 deletion in vasogenic models produces greater brain swelling, higher intracranial pressure (ICP), and increased brain water content, consistent with AQP4 serving as an exit route for excess extracellular fluid [8, 36]. Tait et al. [32] reported the same direction of effect after experimental subarachnoid hemorrhage, where AQP4-null animals cleared fluid less efficiently. Loss of AQP4 also produces brain-wide interstitial fluid stagnation and impaired glymphatic transport on quantitative MRI [37], which provides a mechanistic explanation for the clearance deficit rather than merely restating it (Fig. 2).

**Fig. 2.**
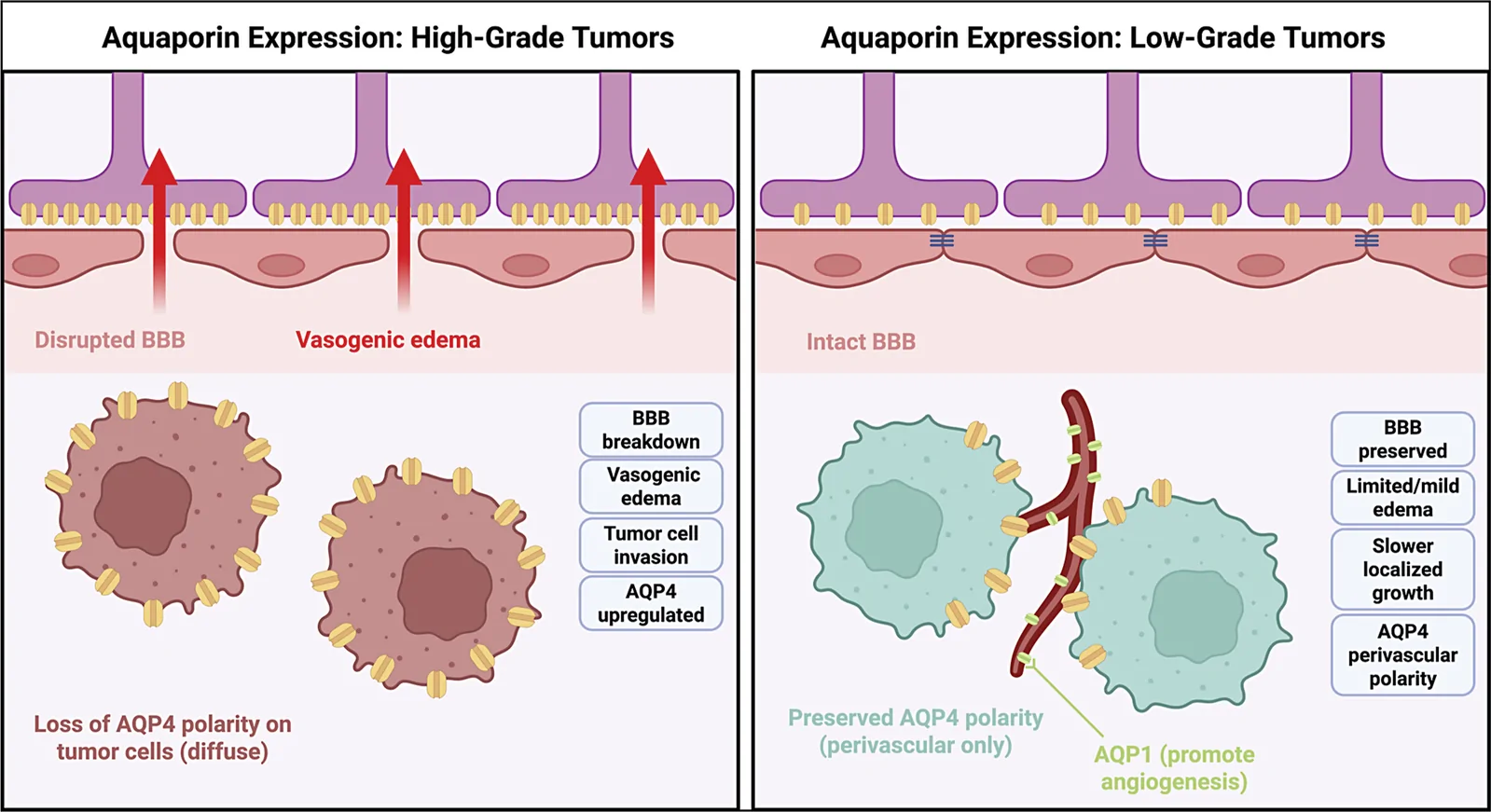
In high-grade tumors, AQP4 is upregulated and loses its normal polarized perivascular distribution, becoming diffusely expressed on tumor cells. This redistribution is associated with BBB disruption, increased vascular permeability, vasogenic edema, and greater tumor invasiveness. Red arrows indicate movement of fluid across the disrupted barrier. In contrast, low-grade tumors generally retain an intact BBB and preserve the predominantly perivascular localization of AQP4, contributing to limited edema and slower, more localized growth. AQP1 expression within the tumor vasculature is depicted as supporting angiogenesis. Yellow membrane channels represent AQP4, green vascular channels represent AQP1, and blue junctional structures indicate preserved endothelial tight junctions.

These two bodies of evidence are frequently cited alongside one another without acknowledging how directly they conflict at the level of therapeutic implication. If AQP4 inhibition is protective in cytotoxic edema but harmful in vasogenic edema, and if brain tumors generate predominantly vasogenic edema, then the most commonly proposed intervention, pharmacologic AQP4 blockade, is being advanced in the setting where the preclinical evidence predicts the least benefit and the greatest potential harm. Three caveats limit how far this objection should be pressed. First, almost none of the vasogenic data derive from tumor models; cortical freeze injury, subarachnoid hemorrhage, and abscess are used as surrogates for a process that in tumors is chronic, spatially heterogeneous, and accompanied by active cell invasion. Second, constitutive knockout animals are not a model of acute pharmacologic inhibition as they undergo developmental compensation, including altered extracellular space volume and increased cortical capillary density. Third, the timing of intervention is almost never varied, although the cytotoxic and vasogenic components of tumor edema are unlikely to peak simultaneously. Resolving whether AQP4 modulation should be directed toward inhibition or toward restoration of polarity, and at what point in the disease course, is the central unanswered question in this field.

A related and underappreciated issue is that in tumor-associated tissue AQP4 expression extends beyond the perivascular end-foot to involve the entire astrocytic membrane [38]. Total AQP4 protein may, therefore, rise while functionally polarized, clearance-competent AQP4 falls (Fig. 3). Studies reporting only aggregate expression cannot distinguish these states, which may account for a substantial portion of the apparent inconsistency between series. Additionally, AQP5 has been implicated in cytotoxic brain edema; Yamamoto et al. [39] demonstrated that hypoxia is a potent stimulus for downregulation of AQP5 in rat astrocytes, although this observation has not been extended to human tumor tissue and its significance remains uncertain.

**Fig. 3.**
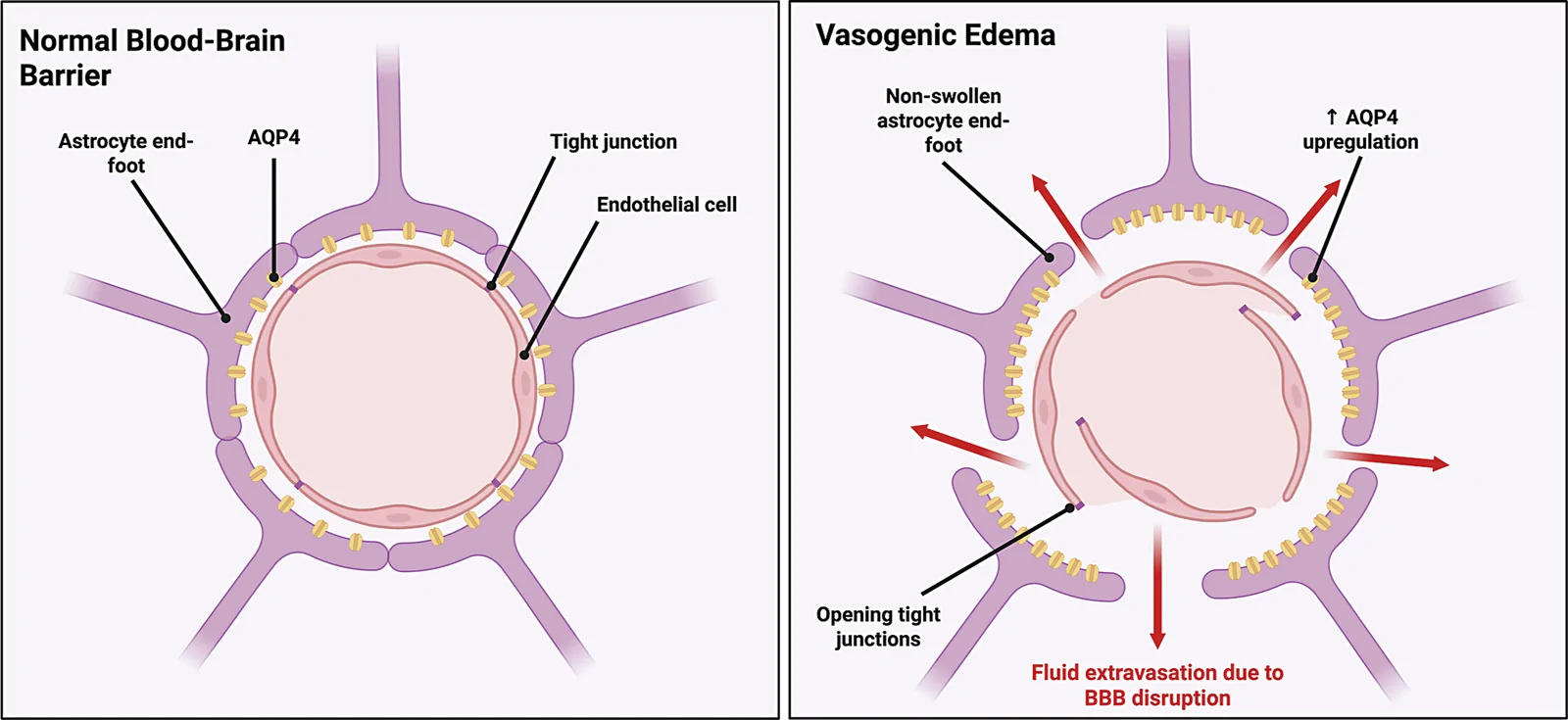
Under normal conditions, cerebral endothelial cells are connected by intact tight junctions and are closely surrounded by astrocyte end-feet, where aquaporin-4 (AQP4) is concentrated along the perivascular membrane. During vasogenic edema, disruption and opening of endothelial tight junctions increase BBB permeability, allowing intravascular fluid to extravasate into the surrounding extracellular space. AQP4 is upregulated along astrocyte end-feet and may facilitate water redistribution after barrier breakdown. Unlike cytotoxic edema, vasogenic edema primarily involves extracellular fluid accumulation rather than marked cellular swelling. Red arrows indicate the outward movement of fluid from the vessel, yellow channels represent AQP4, and purple junctional structures represent endothelial tight junctions.

## AQP Expression across Tumor Types

Immunohistochemical study of human brain tumors first demonstrated upregulation of AQP4 in astrocytes adjacent to edematous tumor tissue [40], and AQP4 has since been implicated in glioblastoma not only in edema formation but also in glymphatic dysfunction, immune evasion, and treatment resistance [41]. In glioblastoma and other high-grade gliomas, Lan et al. [14] reported marked upregulation of AQP4, with overexpression across the entire tumor cell membrane accompanied by loss of perivascular polarity. This redistribution correlates with BBB breakdown and VEGF-driven hyperpermeability and with the development of extensive vasogenic edema. Beyond edema formation, AQP4 has been implicated in tumor cell migration and invasion, and its downregulation has been associated with increased glioblastoma cell apoptosis. Collectively, these findings suggest that in malignant gliomas AQP4 functions both as an amplifier of edema and as a facilitator of tumor motility, a dual role that complicates the therapeutic calculus since suppressing invasion and preserving fluid clearance pull in opposite directions.

By contrast, Faropoulos et al. [15] reported that low-grade gliomas and meningiomas exhibit minimal or focal AQP4 expression, often restricted to perivascular regions or reactive astrocytes rather than to tumor cells themselves. These tumors generally retain a more intact BBB, and the associated vasogenic edema is correspondingly less pronounced than in glioblastoma. In meningiomas, AQP1 expression is more prevalent than AQP4 and is localized primarily to tumor vasculature, where it has been associated with angiogenesis rather than directly with edema generation. Overall, low-grade gliomas tend to preserve AQP polarity and lack the extensive AQP4 redistribution characteristic of high-grade tumors. Excessive AQP4 expression has nonetheless been described in a subset of meningiomas and associated with the peritumoral edema characteristic of those lesions [14, 15], and the discrepancy between series most likely reflects differences in tumor grade distribution, antibody selection, and whether peritumoral rather than intratumoral tissue was sampled. Notably, most of these studies are single-institution, retrospective, and modest in size, and none has been replicated with a prespecified scoring protocol.

## Glymphatic Dysfunction and Imaging-Based Characterization of Peritumoral Edema

The clinical relevance of AQP biology depends on whether AQP4 status can be interrogated in an individual patient before treatment is chosen. Direct measurement requires tissue, and no clinically validated AQP4-specific radiotracer exists. Non-invasive surrogates of perivascular (glymphatic) transport, however, have advanced considerably and are currently the most practical route to translation.

Diffusion tensor image analysis along the perivascular space (DTI-ALPS) estimates water diffusivity along deep medullary veins and has been applied across several brain tumor populations. In 201 patients with glioma, Toh and Siow [42] found that isocitrate dehydrogenase 1 (IDH1) wild-type status and larger peritumoral edema volume were the two independent factors associated with a lower ALPS index, implying that glymphatic impairment tracks with both molecular aggressiveness and edema burden. Subsequent cohorts have reproduced this pattern, showing lower ALPS indices in patients than in controls, an inverse relationship between the ALPS index and both tumor and peritumoral edema volume, and tumor grade emerging as an independent predictor on multivariate analysis. Analogous findings have been reported for meningiomas, where peritumoral edema has been linked to glymphatic dysfunction rather than to microvascular density alone [43].

Most importantly for the present discussion, Gao et al. [44] combined multiparametric MRI with immunofluorescence of resected tissue in 84 patients with glioblastoma, brain metastases, or aggressive meningioma and correlated imaging-derived glymphatic metrics directly with tissue AQP4 expression. This is the closest the field has come to a non-invasive readout of the molecular process this review describes, and it establishes that the imaging surrogate is not merely a correlate of edema volume but is anchored to AQP biology in human tumors.

Several practical applications follow, all of which remain hypotheses requiring prospective testing. First, a pretreatment ALPS index, interpreted alongside conventional FLAIR-based edema volumetry and dynamic contrast-enhanced MRI estimates of vascular permeability, could be used to characterize whether a given patient’s peritumoral signal abnormality reflects predominantly barrier leak or predominantly failed clearance. These two situations have opposite implications for AQP4-directed therapy, and at present they are indistinguishable on routine imaging. Second, serial ALPS measurement offers a candidate pharmacodynamic endpoint for early-phase trials of AQP-modulating agents, which currently lack any biomarker of target engagement in the brain. Third, because glymphatic impairment appears to extend into the contralateral hemisphere and to track with tumor grade and IDH status, it may carry prognostic information independent of edema volume. Fourth, apparent diffusion coefficient and free-water diffusion imaging can partially separate cytotoxic from vasogenic components within the peritumoral region [2], which is directly relevant to selecting between corticosteroid-based and osmotherapy-based management. The principal limitations are that DTI-ALPS is sensitive to region-of-interest placement and head positioning, has not been standardized across vendors, and cannot be reliably computed when tumor or edema encroaches on the periventricular sampling region, precisely the circumstance in which it would be most informative.

## Current Clinical Management of Tumor-Associated Edema

Management of brain tumor-related edema depends on whether cytotoxic or vasogenic mechanisms predominate [7]. In vasogenic edema, corticosteroids remain the mainstay of treatment, acting largely through downregulation of VEGF, stabilization of intercellular tight junctions, and blunting of angiogenic signals that promote permeability. In refractory cases, anti-VEGF therapy may be used as adjuvant treatment to achieve rapid, though often transient, reduction in edema. Surgical debulking can provide benefit by relieving mass effect and reducing production of tumor-derived pro-edematous factors [45]. Osmotic agents such as mannitol are used less commonly in this setting as they provide only temporary relief and do not address the underlying increase in permeability.

In contrast, management of cytotoxic edema differs because the BBB is comparatively intact [7]. The efficacy of corticosteroids is limited, and treatment is directed toward restoring cerebral perfusion and controlling ICP. Hyperosmolar therapy may offer symptomatic relief but should be used judiciously. Emerging approaches such as modulation of AQP4 trafficking remain experimental. Importantly, none of these interventions acts on the water channels themselves; corticosteroids and bevacizumab reduce the delivery of fluid into the interstitium but do not enhance its removal, which is one reason edema recurs promptly on withdrawal and why steroid dependence remains a substantial source of iatrogenic morbidity. Because cytotoxic and vasogenic components overlap in clinical practice, management should be individualized, and no single approach is universally applicable.

## Emerging and Prospective AQP-Targeted Therapies

### Small Molecules and Biologic Agents

Several classes of small molecules, including arylsulfonamides, antiepileptics, and carbonic anhydrase inhibitors, have been identified as potential inhibitors of AQP4-mediated water transport, with acetazolamide demonstrating relatively potent blockade in vitro [22]. Heavy metals such as mercury and zinc and loop diuretics such as bumetanide also inhibit AQP subtypes, but their clinical utility is limited because their edema-reducing effects are likely mediated through alternative pathways, including inhibition of the Na-K-2Cl cotransporter [22]. It is worth noting that the reproducibility of small-molecule AQP4 inhibition has been contested, with several early candidates failing to show activity in independent oocyte and cell-swelling assays, and this history argues for caution in interpreting single-laboratory reports of new inhibitors.

More selective approaches include aquaporumab, a monoclonal antibody directed against AQP4, and the small-molecule antagonists AER-270 and its prodrug AER-271, which have demonstrated specificity for AQP4 and progressed to early clinical evaluation; AER-271 has completed a phase 1 trial, although efficacy data remain pending [20, 23]. Both programs were developed for ischemic and traumatic indications rather than for neoplasia, and neither has been tested in a brain tumor population.

### Co-Targeting of Coupled Ion Channels

Beyond direct AQP4 inhibition, targeting the SUR1–TRPM4 channel complex, which interacts with AQP4 during cytotoxic edema, has shown protective effects. The sulfonylurea glibenclamide reduces sodium influx and cytotoxic swelling and attenuates BBB disruption, brain water content, and contusion volume across multiple rodent models of traumatic brain injury and stroke [22]. This approach has advanced further in clinical development than any direct AQP inhibitor, but again in non-neoplastic indications, and its relevance to chronic tumor-associated edema is assumed rather than demonstrated.

### Gene Silencing and Gene Editing

Genetic approaches provide the strongest preclinical evidence that AQP4 is a tractable target, and also the clearest illustration of why translation has been slow. Genetic deletion of AQP4 reduces ICP, brain water accumulation, astroglial activation, and lesion volume after acute brain injury and AQP4-targeted small interfering RNA in rats attenuates edema, neuroinflammation, and neuronal death [22]. CRISPR/Cas9-mediated knockout has been used to dissect AQP4 function in vivo: Dan et al. [46] combined siRNA silencing with CRISPR/Cas9 editing in a neonatal hypoxic-ischemic model and demonstrated that AQP4 depletion improved long-term neurobehavioral outcome and promoted axonal outgrowth through upregulation of GAP43, indicating effects extending beyond water flux alone. CRISPR/Cas9-generated AQP4 knockout rats have likewise been used to show impaired solute clearance from the CSF compartment, and isoform-specific editing has been employed to demonstrate that the perivascular localization of AQP4, rather than its abundance, governs glymphatic clearance [37].

Translating these tools into therapy for brain tumor-related edema faces four specific obstacles that the current literature tends to elide. First, constitutive germline deletion is a poor model of the intended intervention; a therapeutic agent would need to act acutely and reversibly, whereas knockout animals adapt developmentally, exhibiting expanded extracellular space and increased capillary density that themselves alter edema handling. Second, the therapeutic goal in tumor-associated edema is more plausibly restoration of polarity than reduction of abundance, which favors editing or modulation of the anchoring apparatus, including α-syntrophin and dystrophin-associated proteins, or manipulation of the M1:M23 ratio to influence OAP assembly, over simple gene disruption. Third, delivery must be astrocyte-selective and spatially restricted to the peritumoral rim; adeno-associated viral vectors with astrocyte-specific promoters, such as those based on the GFAP promoter, are the most developed option, but transduction across a partially disrupted blood-tumor barrier is uneven, and this heterogeneity is itself a function of the pathology being treated. Fourth, safety considerations are non-trivial: AQP4 is the target autoantigen in neuromyelitis optica spectrum disorder, so any approach that alters AQP4 conformation, surface presentation, or OAP assembly requires explicit immunologic evaluation, and chronic AQP4 suppression may impair the clearance functions on which the peritumoral brain depends. A realistic near-term research agenda would, therefore, prioritize inducible, astrocyte-restricted, polarity-directed manipulation in orthotopic tumor models with serial imaging endpoints, rather than further replication of constitutive knockout experiments in non-tumor injury paradigms.

## Critical Appraisal, Knowledge Gaps, and Future Directions

Several limitations constrain the conclusions that can currently be drawn, and they are properties of the underlying literature rather than of this review alone. As a narrative review, the search may not have captured all relevant studies, and publication bias favoring positive results in knockout experiments cannot be excluded. Heterogeneity across experimental models, including differences in tumor type, species, injury paradigm, and methods used to assess AQP expression, limits direct comparison between studies. Many mechanistic insights regarding AQP4, AQP1, and other AQPs derive from rodent models or in vitro systems that do not recapitulate the chronicity, spatial heterogeneity, or immune context of the human tumor microenvironment. The roles of specific isoforms, OAP assembly, and channel-interacting proteins such as SUR1–TRPM4 remain incompletely defined and highly context dependent.

Four gaps deserve particular emphasis. First, the field lacks human tumor studies that quantify AQP4 polarity rather than total expression; until perivascular polarization is reported as a primary variable, the apparent contradictions between series cannot be resolved. Second, no study has directly compared AQP4 manipulation across cytotoxic-predominant and vasogenic-predominant phases within the same orthotopic tumor model, which is the experiment required to determine whether inhibition or augmentation is appropriate and when. Third, AQP-targeted agents have no validated biomarker of central target engagement; imaging surrogates of perivascular transport are the obvious candidate but have not been deployed for this purpose. Fourth, clinical data are effectively absent: the agents closest to the clinic were developed for ischemia and trauma, and no AQP-directed therapy has been evaluated in patients with brain tumors.

Priorities for future work follow directly. Prospective human-tissue series should pair quantitative polarity scoring with preoperative multiparametric imaging and edema volumetry, using a prespecified scoring protocol to permit replication. Orthotopic tumor models with inducible, astrocyte-restricted AQP4 modulation should be used to test timing-dependent effects with serial MRI endpoints. Early-phase clinical work should incorporate DTI-ALPS or an equivalent perivascular transport measure as a pharmacodynamic endpoint before efficacy trials are attempted. Finally, given the dual role of AQP4 in edema and in glioma cell migration, therapeutic development should explicitly measure invasion and progression-related outcomes rather than water content alone since an intervention that reduces edema while facilitating infiltration would be a poor trade for the patient.

## Conclusion

AQPs, particularly AQP4, are central regulators of water transport, astrocyte physiology, and edema formation in the tumor-bearing brain. Their dysregulation in high-grade gliomas, metastases, and a subset of meningiomas contributes to both cytotoxic and vasogenic components of edema, amplifying ICP and neurological dysfunction. Insights into AQP4 polarity, OAP assembly, isoform-specific function, and interactions with associated channel proteins have clarified the complexity of AQP biology within the tumor microenvironment, but they have also made clear that AQP4 cannot be characterized simply as pro-edematous. Its net effect depends on barrier integrity, edema phase, and the preservation of perivascular polarization, and this context dependence, rather than any shortage of candidate compounds, is the principal obstacle to therapeutic translation.

Current clinical therapies act indirectly, through modulation of vascular permeability or osmotic gradients, and do not engage the molecular machinery governing water flux. Preclinical evidence supports targeted modulation of AQP4, whether through inhibition, restoration of perivascular localization, or interference with coupled channels such as SUR1–TRPM4, but that evidence remains almost entirely non-neoplastic and largely dependent on constitutive gene deletion. The most tractable next steps are quantitative assessment of AQP4 polarity in human tumor tissue, validation of perivascular transport imaging as a pretreatment stratification and pharmacodynamic tool, and development of inducible, astrocyte-restricted gene-editing strategies aimed at restoring polarity rather than abolishing expression. Progress along these lines, rather than further catalog of candidate inhibitors, is what would convert AQP biology into improved neurological outcomes and quality of life for patients with intracranial neoplasms.

## Conflict of Interest Statement

Michael Karsy reports royalties from Thieme Medical Publishing, consulting from Leica Microsystems, and grant support from JNJ/Ethicon. The other authors have no conflicts of interest to declare.

## Funding Sources

This study was not supported by any sponsor or funder.

## Author Contributions

Y.A.: writing – review and editing, writing – original draft, software, resources, investigation, formal analysis, conceptualization, and data curation. D.B., C.R., and J.K.: writing – original draft, resources, investigation, and data curation. M.K.: writing – review and editing, visualization, validation, supervision, project administration, conceptualization, and methodology. M.P.: writing – review and editing, visualization, validation, supervision, project administration, methodology, investigation, data curation, and conceptualization.
